# The significance of case reports in biomedical publication

**DOI:** 10.4103/0301-4738.67038

**Published:** 2010

**Authors:** Barun Kumar Nayak

**Affiliations:** P. D. Hinduja National Hospital and Medical Research Center, Veer Savarkar Marg, Mahim, Mumbai - 400 016, India. E-mail: editor@ijo.in

Publication in a peer-reviewed journal may be a hobby for some, but it has become a necessity for many due to the new guidelines of Medical Council of India (MCI) for promotion of teachers.[1] Though very often full fledged research is constrained by logistics, clinicians encounter unique cases throughout their practice. Out of the many categories published in peer-reviewed journals, “case reports” are the easiest way to get an article accepted considering that the authors are fully aware of the nitty-gritty of the publication process. This editorial emphasizes the key issues in consideration for accepting a case report for publication. This knowledge will be handy at the time of preparing the manuscript for case reports.

Case reports form an important aspect of publication. They describe important scientific observations which are missed or go undetected in clinical trials. At times rare adverse events can be identified only through case reports. For detecting an event which occurs in 1 in more than 1000 patients, one has to plan a case-control study or cohort study of more than 3000 subjects, which is not practical. It was a case report which linked US Food and Drug Administration (FDA)-approved anorexic agents (fenfluramine and dexfenfluramine) with pulmonary hypertension. This triggered trials evaluating the incidence, mechanism, and risk factor for pulmonary hypertension and finally led to the withdrawal of the drug from the market.[2] The teratogenic effect of thalidomide was also identified through a case report of phocomelia. Sometimes case reports are the first line of evidence for new therapies, as was seen in the role of dapsone in Behcet’s disease[3] or steroids in pemphigus vulgaris.[4] The efficacy of physostigmine in myasthenia gravis was established on the basis of a case report.[5] It is however very difficult to conduct trials for very rare disease or rare indications. An obvious difficulty in recruiting a sizable number of patients to make a powerful study is faced by the author. Furthermore, pharmaceutical companies do not show willingness to sponsor such trials as the potential for revenue is expected to be minimal. Off-label use of drugs is also established by case reports. Thalidomide is an extreme example of this. Its off-label indications are many, some of which were later confirmed by randomized trials.

The cycle of evidence-based medicine is complex. The value of a good case report cannot be undermined. Although in the hierarchy of evidence-based medicine, case reports do not top the list, the hypothesis generated from it is appealing, and can lead to physiological studies followed by clinical trials. The outcome of such clinical trials after favorable interpretation in a clinical context can be incorporated in clinical practice. For a case report to be highly significant, the observations have to be unique and new, the documentation thorough, and the hypothesis logical. The development of acquired immune deficiency syndrome (AIDS), the deadly monster of today, started with a case report of Kaposi’s sarcoma in a young homosexual man published almost 40 years ago.[6] Twenty-four randomization controlled trials were initiated out of the 103 case reports published in the *Lancet* within the next 5 years.[7] This confirms that case reports cannot be ignored.

Case reports have many limitations as well. Sometimes they can raise false alarms. Debendox/Bendectin a combination drug including pyridoxine and doxylamine succinate, was withdrawn from the US market based on case reports citing malformations as it was used to cure morning sickness by pregnant women.[7] However, the drug remained in use in Canada and it did not prove to be teratogenic. Case reports are also subject to bias as 90% report successes as against 10% reporting failures.[8] The methodology of case reports cannot be robust. Most of the once popular but now discarded therapies are based on case reports. Although the readability is high, case reports are not favored by most editors and journals as they are considered less citable articles and do not help in increasing the “impact factor” of the journal. While certain journals do not publish case reports at all, there are journals, which publish only case reports. *The Indian Journal of Ophthalmology* encourages publication of all types of articles.[9]

Just a rare presentation of a case does not justify publication. Editors are always on the lookout to give a new message, which could be in the form of a presentation, management, an outcome, a complication, or an unexplored aspect. The criteria for publishable case reports have been extensively enumerated by Cohen.[2] If the case that you want to report fulfills any criterion out of the extensive list provided, you must think of publishing the particular case at hand. Cohen[2] has also provided elaborate guidelines for writing case reports which must be read by all before writing a case report to increase the chances of acceptance of their report.

Case reports are beneficial for authors too. For beginners, a case report provides a learning opportunity in the practice of scientific writing. However, it should be kept in mind that though they are easy to write, they should not be taken lightly. A quick peep into the review process and the guidelines that a reviewer follows will give you some useful hints in writing a good case report. The introduction section should clearly and adequately explain the rationale for publication. All statements and claims should be substantiated by references. Introduction should be prepared like that of a lawyer defending/pleading for justification of publication supported by the available literature. It should be so strong that the editor should have no choice but to be convinced regarding the importance of the paper. The case description should be adequate, brief, and clear. The results of the investigations should be properly described and the results of less common laboratory investigations should be accompanied by normal values. Above all, it has to be documented properly and completely. The authors must also remember to protect the identity of the patients in photos and all imaging, not forgetting to obtain a no-objection letter from them before publication. Lastly, one should keep in mind that the editors expect affirmative answers on the following questions from the reviewer. (i) Is there adequate evidence to support the author’s diagnosis? (ii) Is there adequate evidence to support the author’s recommendation? (iii) Are other plausible explanations considered and refuted? (iv) Are the implications and relevance of the case discussed? (v) Do the authors indicate direction, for future investigations or management of similar cases? (vi) Is a hypothesis generated?

The value of a case report can be judged by considering the following five aspects:

- Documentation
- Uniqueness
- Educational value
- Objectivity
- Interpretation

An elaborate scoring system of 0–2 for each of the above aspects has been well described by Pierson.[10] Out of a cumulative score of 0–10, a case report scoring 9 or 10 points can be considered as a valuable addition to the literature. And while a score of 6–8 should be cautiously interpreted, articles with a score of 5 or less should be rejected.

The publishable case reports should be thoroughly investigated, properly managed, and meticulously documented. It should be written well highlighting the unique features with a logical generation of a hypothesis. I am sure this editorial will be helpful in preparing the manuscript for case reports which the editors will find difficult to reject.
